# Bovine pericardial versus porcine bioprosthetic valves in pulmonary position for adult congenital heart disease: A three-center, retrospective, propensity-weighted analysis of clinical outcomes

**DOI:** 10.1016/j.ijcchd.2026.100700

**Published:** 2026-08-05

**Authors:** Eliú David Pérez Nogales, Elisabet Viera Reyes, Stefano Urso, María Luz Polo López, Blanca Torres Maestro, Jesús María González-Martín, Pablo Meras Colunga, Jose Ruíz Cantador, Carlos Merino Argos

**Affiliations:** a Complejo Hospitalario Universitario Insular–Materno Infantil, Department of Cardiology, Las Palmas de Gran Canaria, Spain; b Hospital Universitario Dr. Negrín, Department of Cardiac Surgery, Las Palmas de Gran Canaria, Spain; c Hospital Universitario La Paz, Departments of Cardiac Surgery, Madrid, Spain; d Hospital Universitario Dr. Negrín, Methodology and Biostatistics Unit, Las Palmas de Gran Canaria, Spain; e Hospital Universitario La Paz, Departments of Cardiology, Madrid, Spain

## Abstract

**Introduction and objective:**

Adults with congenital heart disease affecting the right ventricular outflow tract often require repeated pulmonary valve replacements (PVR) throughout life, making prosthesis durability a critical determinant of long term outcomes. Bovine pericardial and porcine bioprosthetic valves are the most widely used in surgical PVR, yet comparative durability data remain inconsistent, partly due to reliance on nominal valve size rather than functional parameters. This study aimed to compare long term durability between both valves using indexed effective orifice area (iEOA) and analytic methods focused on prosthesis related performance.

**Methods:**

We conducted a retrospective, multicenter observational study including pediatric and adult patients undergoing surgical PVR between 2013 and 2024 at three tertiary hospitals in Spain. The primary endpoint was a composite outcome of all cause mortality, reintervention, and structural valve deterioration. Propensity scores were constructed using clinically selected covariates and applied through inverse probability weighting (IPW). To isolate prosthesis related durability, survival was analysed by Cox regression model with left truncation at 1 year.

**Results:**

A total of 231 patients were included (49.4% female; 64.1% with tetralogy of Fallot). Overall, 19.0% experienced a MACE during follow up, occurring at a median of 4.8 years after the truncation point. After IPW adjustment, baseline characteristics were well balanced. In the IPW adjusted Cox regression model, porcine prostheses were associated with a lower hazard of MACE (HR 0.37; 95% CI 0.16–0.83; p = 0.015).

**Conclusions:**

When early postoperative events are excluded and baseline differences minimized, porcine bioprostheses exhibit superior mid and long term durability compared with bovine pericardial valves.

## Graphical abstract

**Figure:**
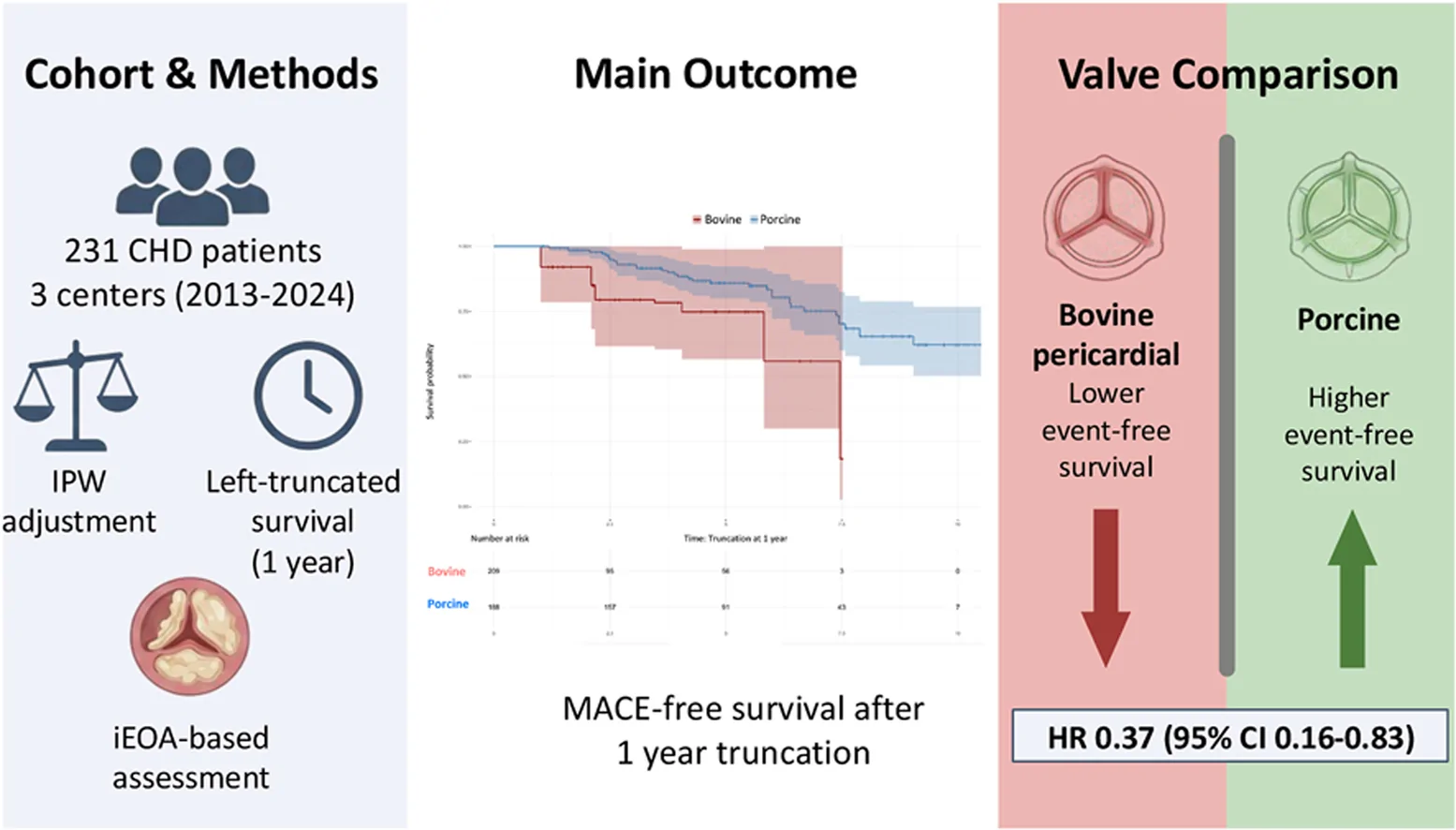

## 1 Introduction

Surgical correction of congenital heart disease involving significant right ventricular outflow tract (RVOT) obstruction—such as tetralogy of Fallot (ToF)—has markedly improved long-term survival into adulthood but imposes a high lifetime requirement for pulmonary valve replacement (PVR) [1,2]. Optimal prosthesis selection is therefore critical in this predominantly young patient population to minimize the cumulative burden of reinterventions over time [3,4].

Bioprosthetic surgical valves—either bovine pericardial or porcine tissue—are the preferred option in the pulmonary position due to their ready availability, favorable hemodynamic profile, and lack of mandatory long-term anticoagulation, as well as their facilitation of potential future transcatheter interventions [3,4]. The American Heart Association recognizes bovine pericardial models (CE PERIMOUNT Magna®, Magna Ease®, Inspiris Resilia®, -Lifesciences Corp., Irvine, CA, USA-) and porcine-mounted valves (Hancock II®, Mosaic® -Medtronic, Minneapolis, MA, USA-, Epic®, Epic Plus® - Abbott, Minneapolis, MN-) as the most widely utilized, citing their durability and procedural versatility in surgical PVR (SPVR) [2]. Homografts remain an important alternative for PVR, particularly in younger patients. Cocomello et al. found that pulmonary homografts were associated with significantly lower risk of reintervention or structural valve degeneration compared with bioprostheses (adjusted HR 0.27; 95% CI 0.13–0.55; p < 0.001) at 9-year follow-up [5]. Bell et al. reported that in patients aged 10–20 years, bioprostheses had nearly 10 times the risk of structural valve degeneration compared with homografts [6]. However, homograft availability is limited.

Durability remains a pivotal concern. Multicenter analyses report that structural valve degeneration typically emerges between 10 and 15 years post-implant, with considerable variability influenced by prosthesis type and patient factors [3,4,7]. In a cohort of 482 adults with congenital heart disease, the 5-year cumulative incidence of significant valve dysfunction was higher for bovine pericardial prostheses (39%) compared to porcine valves (17%), although overall survival did not differ between groups [4]. A retrospective series of 258 cases further demonstrated lower rates of reoperation and long-term valve failure with porcine bioprostheses (hazard ratio for reoperation, 2.17; 95% CI, 1.26–3.72; p = 0.005) [3], whereas a single-center analysis of 170 patients found no significant durability differences after adjustment for age and valve size [8]. However, a major limitation in comparing durability across bioprosthetic types is the frequent reliance on nominal valve size, a metric that does not adequately capture the functional performance of the implanted prosthesis. Differences in stent design, leaflet configuration, and patient anatomy may lead to substantial discrepancies between labelled size and actual hemodynamic performance. Contemporary evidence in valve replacement research has shown that functional parameters, such as the effective orifice area (EOA), particularly indexed to body surface area (iEOA), correlate more closely with postoperative gradients, prosthesis–patient mismatch, and long-term outcomes than the labelled size alone [9]. Because EOA reflects the actual flow capacity of the implanted valve—integrating design characteristics, leaflet configuration, and patient-specific anatomy—it offers a more physiologically meaningful metric for comparative analyses. For this reason, we used iEOA rather than nominal size to evaluate prosthetic performance in our cohort.

More recent attention has focused on the Inspiris Resilia bovine pericardial model, which has been showed to increase early prosthetic dysfunction rate and an increased incidence of endocarditis compared with other bovine and porcine bioprostheses [10,11]. Although data on endocarditis in pulmonary SPVR remain limited, both bovine and porcine surgical bioprostheses appear to confer lower infective risk than bovine jugular vein conduits [7,11,12].

In this context, the present study compares the durability and reintervention rates of surgical bovine pericardial versus porcine bioprosthetic valves in the pulmonary position among adults with congenital heart disease.

## 2 Methods

This retrospective, three-centre observational study included paediatric and adult patients with congenital heart disease who underwent SPVR between January 2013 and January 2024 at three tertiary-care hospitals in Spain. Eligible subjects were identified by each site's principal investigator from surgical logs and institutional databases. Patients were required to have received a bioprosthetic pulmonary valve—either bovine pericardial or porcine tissue—. A total of 10 patients were excluded from the analysis because they presented more than 20% missing values, which precluded reliable endpoint assessment. Homografts were not included in the present analysis because their use in the participating centers was limited during the study period. Their exclusion from our cohort reflects real-world practice in the participating centers. Nonetheless, homografts remain an important alternative in selected anatomical scenarios.

The primary composite endpoint was the occurrence of any of the following: all-cause mortality, surgical or percutaneous reintervention on the pulmonary valve or conduit, and structural valve deterioration—defined as at least moderate pulmonary stenosis (peak velocity ≥ 3.5 m/s or peak gradient ≥ 49 mmHg) or at least moderate regurgitation. Regurgitation severity was graded according to American Society of Echocardiography guidelines [13], incorporating quantitative and qualitative measures (jet width, Doppler velocities, pressure half-time, deceleration time, and holodiastolic flow reversal). Secondary analyses examined each endpoint component separately.

Echocardiographic follow-up was performed in all participating centers according to structured institutional protocols, broadly aligned with European adult congenital heart disease guidelines. This included a transthoracic echocardiogram immediately after implantation, annual imaging thereafter, and semi-annual studies once significant obstruction or regurgitation was detected. All examinations were reviewed during MACE adjudication, including serial studies before and after the first image meeting MACE criteria. However, the full longitudinal dataset—typically 10–11 echocardiograms per patient over long-term follow-up—was not incorporated into the database, as it exceeded the scope of the present analysis. Importantly, surveillance frequency did not differ between prosthesis groups, minimizing the risk of differential detection bias. Cases with borderline (mild–moderate) dysfunction were flagged for centralized review: the principal investigator re-evaluated archived digital images via a structured template based on predefined thresholds, and, if image quality or interpretation remained uncertain, a second congenital cardiac imaging expert adjudicated the findings. For statistical analysis, the first qualifying echocardiographic diagnosis of valve dysfunction was used as the index MACE event, even when subsequent reintervention or death occurred, ensuring consistency, avoiding double counting, and capturing the earliest clinically meaningful manifestation of prosthesis deterioration.

All readings, including measurement date and final classification, were recorded in a secure Microsoft Access database to ensure traceability. Data collection complied with the ethical standards and approval requirements of the institutional review boards of the participating hospitals. In addition, all patients—or their legal guardians in the case of minors—provided written informed consent for the use of their clinical information for research purposes.

Clinical and procedural data were abstracted from electronic health records into a relational database organized into linked tables for demographics and congenital diagnosis, PVR procedure details (prosthesis type, material, and size), and long-term outcomes (mortality, reintervention, echocardiographic dysfunction, and major adverse cardiovascular events). For the purposes of statistical analysis, we used the indexed body surface effective orifice area (iEOA) instead of the nominal prosthesis size. Prior studies have demonstrated that labelled valve size does not reliably represent the functional opening area of a bioprosthesis, whereas EOA provides a direct measure of its hemodynamic performance [9]. Because EOA better captures inter-prosthesis variability related to design, stent geometry, and leaflet configuration, it is considered a more accurate parameter for assessing prosthesis–patient interaction. Accordingly, iEOA was selected as the primary valve-related continuous variable in all comparative analyses. Reference EOA values were obtained from the systematic compilation by Dismorr et al. [9]. As no published EOA reference values exist for surgical bioprostheses specifically in the pulmonary position — a gap acknowledged by both the EACTS/STS/AATS Valve Labelling consensus — values derived from the aortic position were used as the best available approximation.

To maintain confidentiality, each patient was assigned a unique alphanumeric study identifier, with the linkage to medical record numbers stored separately under restricted access.

All analyses were conducted in R (R Core Team, 2024; v4.4.2). Descriptive statistics for continuous variables include mean ± SD and quartiles (25th, median, 75th percentiles), with distributional normality tested by the Kolmogorov–Smirnov test; categorical variables are presented as counts and percentages. Between-group comparisons employed Student's t-test or Mann–Whitney *U* test for continuous data and χ^2^ or Fisher's exact test for categorical data.

Variables included in the propensity score model were selected a priori based on clinical relevance and established associations with both prosthesis type and long-term outcomes. In accordance with methodological recommendations for propensity score construction [14], no automated variable selection procedures (e.g., stepwise regression, AIC-based selection, or significance-driven inclusion) were applied. Instead, covariates included in the propensity score model (sex, age at surgery, body mass index, effective orifice area indexed to body surface area (iEOA), and underlying congenital heart disease diagnosis [tetralogy of Fallot vs other congenital heart disease]) were selected a priori based on their clinical relevance and on previous studies and clinical registries in congenital heart disease, which have identified these factors as predictors of structural valve deterioration, mortality, or other adverse outcomes in this setting [4,15,16]. Propensity scores obtained from this model were then used to calculate inverse-probability weights (IPW), assigning weights of 1/(1 – PS) for bovine and 1/PS for porcine recipients, to balance baseline characteristics between groups. Covariate balance after weighting was assessed via standardized mean differences (SMD), with an absolute SMD < 0.15 indicating adequate balance. After IPW adjustment, baseline characteristics were well balanced (all SMDs < 0.15 except for age – 0.151), demonstrating successful mitigation of selection bias. Propensity score distributions and SMD plots are provided in Supplementary Material, Section 4**. Propensity Score. Figures 3 and 4**.

To specifically evaluate prosthetic durability rather than early postoperative risk, survival analyses were performed using a Cox regression proportional hazards model with left truncation at 1 year. Early mortality after pulmonary valve replacement is predominantly influenced by perioperative factors and patient comorbidities, and does not reflect the intrinsic performance or structural integrity of the bioprosthesis [17,18]. By excluding events occurring within the first postoperative year from the risk set, the analysis focuses on the period during which structural valve degeneration and material-related failure mechanisms become clinically relevant. This approach provides a more accurate assessment of long-term prosthetic durability and minimizes bias introduced by early, non–prosthesis-related events [17,18].

Postoperative antithrombotic/anticoagulation therapy was not standardised across the participating centers. Aspirin was commonly used at the discretion of the operating surgeon—typically for 3–6 months—although long-term continuation varied over time and between institutions. Because early postoperative antithrombotic regimens were not uniformly documented across the study period, detailed longitudinal data on drug type, dose, and adherence could not be consistently retrieved.

## 3 Results

A total of 231 patients met inclusion criteria. The cohort showed a balanced sex distribution (49.4% female), with tetralogy of Fallot representing the most common underlying diagnosis (64.1%). Overall, 19.0% experienced a MACE during follow-up. The mean age at surgery was 33.7 ± 15.7 years, and the average iEOA was 1.07 ± 0.29 cm^2^/m^2^.

When comparing prosthetic materials, 37 patients (16%) received bovine and 194 (84%) received porcine bioprostheses. Baseline characteristics were largely similar between groups. Sex distribution did not differ (female 51% vs. 49%; p > 0.9), nor did the prevalence of tetralogy of Fallot (57% vs. 65%; p = 0.4). Prior surgical history was also comparable, with similar proportions of patients having undergone only one previous intervention (48% vs. 52%; p = 0.8). Body mass index and age at intervention were likewise balanced (both p > 0.3).

Two variables demonstrated statistically significant differences. First, the distribution of prosthetic sizes differed markedly between groups (p < 0.001), with bovine valves predominantly implanted in smaller labelled sizes (25 mm in 68% of cases), whereas porcine prostheses were more frequently 27–29 mm (79% of implants). However, iEOA values were higher in the bovine group compared to the patients who received porcine prostheses (mean iEAO value 1.22 ± 0.42 vs. 1.04 ± 0.25 respectively; p = 0.014), reflecting a larger functional orifice area relative to body surface area. No other baseline variable showed significant between-group differences. Table 1.

**Table 1:** Baseline characteristics.

Variable	Bovine n = 37^a^	Porcine n = 194^a^	P-value
**Sex**			>0.9
**Male**	19 (51%)	95 (49%)	
**Female**	18 (49%)	99 (51%)	
**Baseline cardiac contidion**			0.041
**mean (sd); median (q1, q3)**	3.54 (2.89); 2.00 (1.00, 5.00)	2.59 (2.49); 1.00 (1.00, 5.00)	
**ToF (Tetralogy of Fallot)**			0.4
**No**	16 (43%)	67 (35%)	
**Yes**	21 (57%)	127 (65%)	
**Previous surgeries**			0.8
**One**	15 (48%)	96 (52%)	
**Two or more**	16 (52%)	88 (48%)	
**BMI (body mass index)**			0.6
**mean (sd); median (q1, q3)**	23.0 (6.4); 23.0 (17.0, 27.4)	23.5 (5.1); 23.7 (20.1, 27.2)	
**Nominal prosthesis size (mm)**			<0.001
**21**	2 (5.4%)	2 (1.0%)	
**23**	9 (24%)	7 (3.6%)	
**25**	25 (68%)	33 (17%)	
**27**	1 (2.7%)	79 (41%)	
**29**	0 (0%)	73 (38%)	
**Valve type**			
**Mosaic®**		**192**	
**Biocor®**		**2**	
**Magna edwards®**	**2**		
**Magna edwards ease®**	**9**		
**Perimount edwards®**	**9**		
**Inspiris reselia®**	**17**		
**Age at intervention**			0.3
**mean (sd); median (q1, q3)**	31 (17); 39 (15, 45)	34 (15); 37 (19, 46)	
**IEOA**^b^			0.014
**mean (sd); median (q1, q3)**	1.22 (0.42); 1.09 (0.93, 1.24)	1.04 (0.25); 0.97 (0.89, 1.10)	

a n (%) = number (percentage).

b Indexed to body surface effective orifice area.

A considerable proportion of MACE events occurred in patients implanted with smaller prostheses (<25 mm), consistent with the well-established association between small valve size and accelerated structural valve deterioration. In the porcine cohort, 5 of 37 MACE events involved valves below 25 mm, all in patients under 30 kg. In the bovine cohort, 3 of 7 MACE events occurred in valves below 25 mm, again predominantly in patients under 30 kg. Survival analysis confirmed significantly reduced durability in smaller prostheses (p = 0.0059 overall; p = 0.0031 in porcine valves). Given that prosthesis size is one of the strongest determinants of bioprosthetic longevity, and that smaller valves are more frequently implanted in porcine recipients, adjusting for valve size through propensity weighting is essential to isolate the true effect of material on durability. **See** supplementary material. Section 1.

Before applying left truncation, a total of 44 MACE events were recorded in the overall cohort, including 37 in the porcine group and 7 in the bovine group. As detailed in Table 2, there were no significant differences in the incidence or distribution of MACE between groups at this stage. After applying left truncation at 1 year to isolate prosthesis-related durability, 193 patients contributed to the survival analysis. The median follow-up from the truncation point was 4.8 years (IQR 3.0–6.7), with a maximum of 10.7 years, during which 36 patients (18.7%) experienced a MACE.

**Table 2:** Follow-up events. Of the six deaths that occurred in the porcine group, four took place in the immediate postoperative period (two due to gastrointestinal bleeding, one due to respiratory distress, and one due to hepatic failure). The remaining two deaths were related to oncologic disease (lung cancer and cholangiocarcinoma).

Variable	Bovine n = 37^*1*^	Porcine n = 194^*1*^	P-value
**Mace**			>0.9
**No**	30 (81%)	157 (81%)	
**Yes**	7 (19%)	37 (19%)	
**All cause mortality**	0	6	
**Structural valve deterioration**	6	31	
**Valvular regurgitation**	2 (33.3%)	22 (71%)	
**Valvular stenosis**	1 (16.6%)	8 (25.8%)	
**Mixed valvular disease**	3 (50%)	1 (3.2%)	0.004
**Reintervention (valvular lesion not specified)**	1	0	

**Fig. 1:**
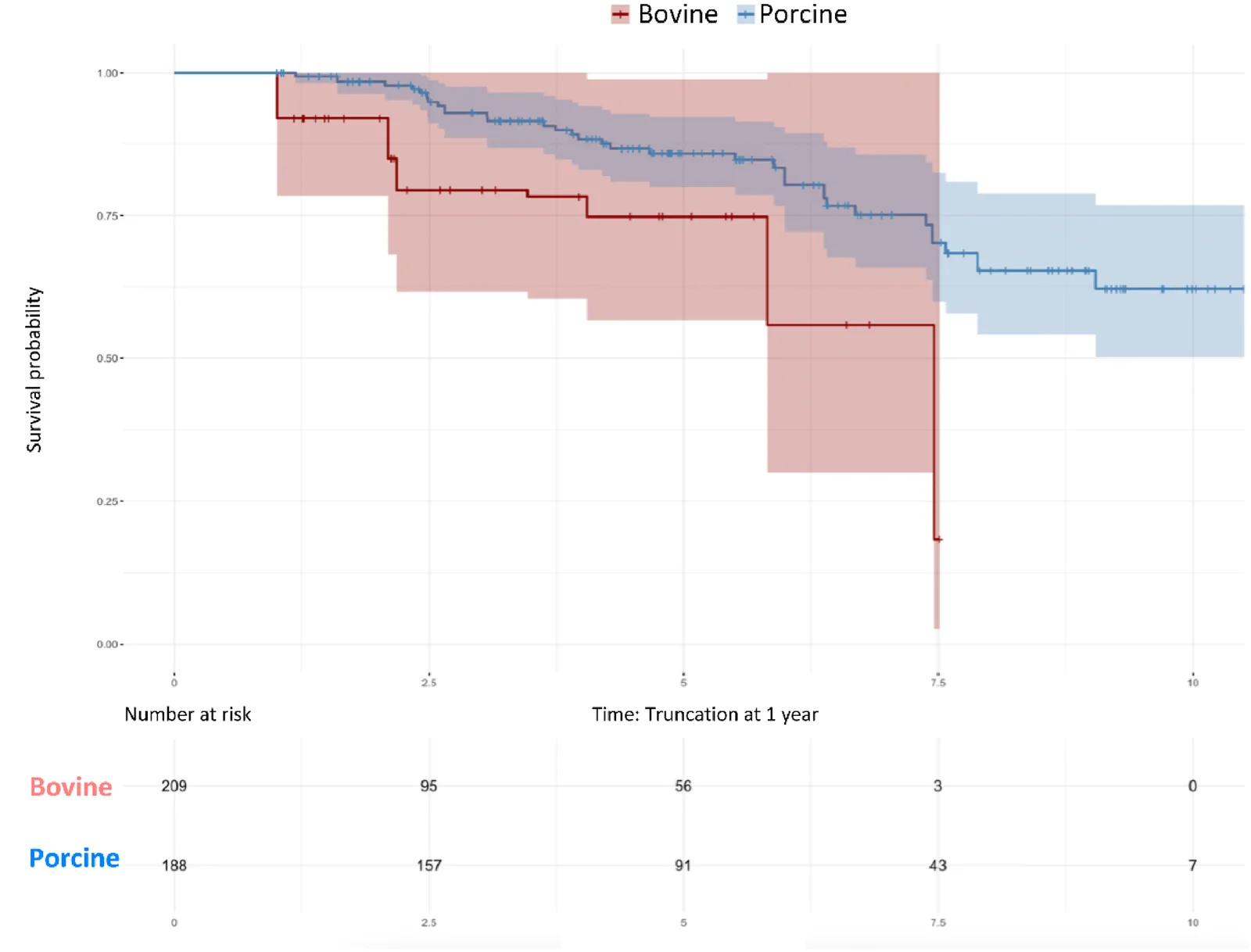
(Central Image): Inverse probability–weighted Kaplan–Meier curves, truncated at 1 year, illustrating long term event free survival for bovine versus porcine bioprostheses. * Number at risk: The number at risk shown in the figure corresponds to the weighted sample size after inverse probability weighting, rather than the raw cohort count; this is standard in propensity-adjusted survival analyses.

Weighted Kaplan–Meier (Fig. 1) curves demonstrated a clear and progressive separation between groups, with porcine prostheses showing consistently higher event-free survival compared with bovine valves throughout mid- and long-term follow-up. In the IPW-adjusted Cox proportional hazards model, porcine prostheses were associated with a significantly lower hazard of MACE (HR 0.37; 95% CI 0.16–0.83; p = 0.015). Model diagnostics supported the robustness of this association, with a concordance index of 0.63 and highly significant likelihood ratio and score tests (p < 0.0001). These findings indicate that, once early postoperative risk is excluded and baseline characteristics are balanced through inverse probability weighting, porcine bioprostheses demonstrate superior long-term durability compared with bovine valves.

## 4 Discussion

The present multicenter, retrospective cohort study provides a comprehensive evaluation of long-term outcomes following surgical pulmonary valve replacement (PVR) with bovine pericardial and porcine bioprosthetic valves in a diverse population of children and adults with congenital heart disease. By applying left truncation at one year and inverse probability weighting to isolate prosthesis-related durability from early postoperative risk, our analysis revealed a significant advantage in long-term event-free survival for porcine bioprostheses.

### 4.1 Comparative long-term outcomes and durability

Although our findings indicate superior long-term durability for porcine bioprostheses, the existing literature presents a more nuanced picture, with studies both supporting and challenging this observation. Patel et al. reported similar 5-year survival rates for both valve types in a cohort of 482 congenital heart disease patients, with no significant difference in overall valve failure rates, although bovine pericardial valves exhibited a higher incidence of valve dysfunction at mid-term follow-up [4]. Chen et al. found no significant difference in the risk of reintervention by valve type in a single-center series of 170 patients, with both groups demonstrating good intermediate-term outcomes and quality of life [8]. Egbe et al., in a large Mayo Clinic cohort, observed no difference in prosthesis longevity by valve type, with a 15-year cumulative incidence of prosthetic valve dysfunction approaching 50% for all bioprosthetic valves, underscoring the limited durability of these devices and the need for individualized valve selection and lifelong surveillance [7].

Nevertheless, not all evidence aligns with these observations, and multiple investigations have identified significant disparities in long-term performance between the two types of bioprosthesis. Kwak et al., identified a higher reoperation rate and greater prosthetic valve dysfunction in patients receiving bovine pericardial valves compared to porcine valves, particularly in younger patients and those with congenital anomalies [3]. This finding is echoed in a nationwide population-based study (with a total of 1295 patients enrolled) by Lim et al., which found a trend toward lower reoperation rates in the porcine group compared to the bovine group, although the difference did not reach statistical significance after propensity matching [1].

The American Heart Association, in its 2024 scientific statement [2] on the long-term management of right ventricular outflow tract (RVOT) dysfunction in repaired tetralogy of Fallot, notes that the performance and durability of different types of biological valves and conduits appear to be relatively similar, although some studies suggest that certain bovine pericardial valves, such as the Mitroflow [19], may be associated with worse durability. The AHA statement underscores the importance of individualized valve selection based on patient characteristics and a lifelong strategy aimed at minimizing reoperations, with particular emphasis on optimizing valve hemodynamics to reduce the long-term risk of reintervention [2]. In line with this perspective, such decisions are best made within a multidisciplinary Heart-Team, ensuring that the chosen approach aligns with both the patient's current status and their anticipated lifetime management needs.

Although the present study focuses primarily on surgical valve replacement, it is important to briefly consider how these findings fit within the broader landscape of lifetime valve management, which increasingly includes transcatheter options. In our clinical experience, surgical replacement often represents a reasonable initial step for many patients, as it may offer greater durability and help preserve an optimal landing zone for future transcatheter interventions if required. Conversely, initiating the treatment sequence with a transcatheter valve can, in some cases, make subsequent surgical procedures more challenging due to the presence of prior stents [20].

### 4.2 Modes of failure and pathological considerations

The present study's findings regarding early and late event rates are further contextualized by recent investigations into the modes of failure of bioprosthetic valves. Keshishi et al. compared explanted porcine and bovine pericardial bioprosthetic valves and found that the primary mode of failure was regurgitation in porcine valves (often due to cusp tears) and stenosis in bovine pericardial valves (primarily due to calcification) [21]. This distinction in failure modes may inform surgical decision-making, particularly in patients with specific anatomical or hemodynamic considerations. Grunkemeier et al. similarly reported that leaflet tear was the predominant mode of structural valve deterioration in porcine valves, while fibrosis and calcification were more common in pericardial valves [22]. These findings suggest that while overall durability may be similar, the clinical manifestations and management of valve failure may differ by prosthesis type. In our study, the distribution of failure modes differed significantly between porcine and bovine bioprostheses. Among porcine valves, failure was predominantly due to regurgitation, whereas bovine valves showed a higher relative proportion of mixed lesions and stenosis (p ≈ 0.004).

### 4.3 Patient selection and prosthesis choice

The multivariable analysis in the current study identified stenotic indication as strong predictors of porcine valve implantation, while higher body-mass index (BMI) modestly favored bovine pericardial valve use. These selection patterns likely reflect surgeons’ preferences for the mechanical performance, sewing ring flexibility, and radial strength of porcine tissue in tight RVOT conduits, contrasted with the hemodynamic profile and potential for percutaneous valve-in-valve interventions offered by bovine pericardial designs, especially in patients with higher BMI or anticipated future interventions. The American Heart Association recommends tailoring valve choice to patient age, anatomy, and the potential for future transcatheter interventions, with stented bioprostheses providing a suitable landing zone for future valve-in-valve procedures [2], as the SCAI/AATS/ACC/STS consensus statement also highlights [23].

### 4.4 Short-term outcomes and early valve dysfunction

Multiple large series and cohort studies have shown that both valve types are associated with low perioperative mortality and morbidity, with no significant differences in early postoperative complications or operative mortality between groups.

Early MACE rates are low for both valve types, and freedom from reintervention at one year typically exceeds 95% in contemporary cohorts [1], reaching up to 99% in some series [8]. In our study cohort, bovine pericardial valve recipients had 100% MACE-free survival at one year, whereas porcine valve recipients showed a slightly lower rate (95.8%); however, this difference was not statistically significant after adjustment for baseline risk. This pattern is consistent with other studies, which report that early reintervention and valve dysfunction are rare events in the first postoperative year for both valve types. Egbe et al. reported a cumulative incidence of significant valve dysfunction (mean gradient ≥35 mmHg or moderate PR) of 7.5% at one year, without material-specific differences [7]. Lim et al.’s nationwide Korean cohort (n = 1032; mean age 29 years) found one-year freedom from reintervention of 97.8% overall, with bovine vs. porcine 1-year freedom rates of 98.1% vs. 97.5% (p = 0.42) and similar survival at 24 months [1].

Regarding valve dysfunction, both porcine and bovine pericardial valves maintain excellent hemodynamic performance in the first year, with very low rates of moderate or severe regurgitation or stenosis. No significant differences in early valve dysfunction have been consistently observed, and the risk of early reintervention is minimal for both groups.

Although early reintervention is uncommon, caution is warranted because certain bioprostheses have demonstrated suboptimal early (and late) performance, particularly the Mitroflow® and, more recently, the Inspiris Resilia®. This consideration is especially relevant to our study, in which 45.8% of bovine pericardial valves were Inspiris, underscoring the need to interpret our results within the context of prosthesis-specific performance. For the Mitroflow® bovine pericardial valve, early outcomes are generally acceptable and similar to other tissue valve options, with low perioperative morbidity and no early mortality reported. Freedom from moderate or greater pulmonary insufficiency at one year is high (over 80%), and freedom from significant stenosis or reintervention is also favorable. No cases of premature structural deterioration have been observed in the first year, and early reintervention rates are low, comparable to other bioprosthetic valves in the pulmonary position [24].

In contrast, the Inspiris Resilia valve has demonstrated concerning short-term durability in the pulmonary position. Multiple studies report a high incidence of early prosthetic regurgitation, particularly when the valve is implanted in the native right ventricular outflow tract. Within the first year, up to 22% of patients developed moderate regurgitation and 11% developed severe regurgitation, with some requiring transcatheter valve-in-valve procedures. Early freedom from valve failure and moderate or greater regurgitation is significantly lower for Inspiris compared to standard porcine valves, such as the Mosaic. These findings suggest that the Inspiris Resilia valve may be less suitable for the pulmonary position, especially in native outflow tracts [25,26].

Given these concerns, we specifically evaluated the performance of Inspiris valves within our cohort. Of the 37 bovine prostheses implanted, 17 were Inspiris models (45.9%). Three of these experienced MACE (17.6%), compared with four events among the 20 non-Inspiris bovine valves (20%). In contrast to previously published reports, Inspiris valves in our dataset did not exhibit a disproportionately higher event rate. This discrepancy may reflect the relatively small sample size of Inspiris valves in our cohort, and larger studies may reproduce the early-failure signal described in the literature. Nonetheless, the absence of an increased MACE burden in our Inspiris subgroup supported their inclusion in the main analysis to avoid introducing selection bias. A detailed comparison between Inspiris and non-Inspiris bovine valves is provided in **Supplementary Material,** Section 2.

### 4.5 Strenghts and limitations

Key strengths of our investigation include the multicenter design, extended follow-up that provides a robust window to evaluate true prosthesis-related durability (up to 12 years). Echocardiographic endpoints were systematically adjudicated using predefined criteria and central review, ensuring measurement consistency. Another important strength of this study is the use of iEOA rather than nominal prosthesis size to characterize valve performance. Nominal diameters often fail to reflect the true functional opening of a bioprosthesis, as they do not account for differences in stent geometry, leaflet configuration, or patient-specific anatomical constraints. By relying on iEOA—a physiologically grounded parameter that integrates actual flow capacity relative to body surface area—we were able to compare prostheses on a functionally meaningful basis and minimize misclassification related to labelled size.

Methodologically, the analysis benefits from rigorous adjustment using inverse probability weighting, which effectively balances baseline characteristics and minimizes confounding arising from clinical selection of valve type. The choice of covariates was grounded in clinical reasoning and contemporary guideline recommendations, ensuring that the weighting model reflected meaningful determinants of prosthesis performance.

Early postoperative events were analyzed separately to avoid conflating perioperative complications with true prosthesis-related durability in accordance with VARC-3 recommendations [27]. We applied a one-year left truncation a priori, before reviewing any event distribution, specifically to prevent selection bias and ensure that durability assessment reflected intrinsic bioprosthetic performance rather than early outcomes driven by surgical risk, comorbidities, or procedural complications. Together, these features strengthen the validity and interpretability of our findings. Within this first year, only 8 MACE events occurred (3.46% of the cohort), of which 6 were clearly unrelated to valve performance—gastrointestinal hemorrhage, respiratory failure, hepatic decompensation, or malignancy—while only 2 represented true prosthetic valve failure. All early events were observed in the porcine group, a finding attributable not only to the markedly larger sample size but also to a potential channeling bias: porcine prostheses were more frequently selected in anatomically or clinically complex patients, partly reflecting greater surgeon familiarity and confidence with these valves due to their predominant use over the years. This selection pattern would naturally concentrate perioperative complications in the porcine cohort irrespective of intrinsic prosthesis performance. Including these early events in the primary analysis would have introduced substantial confounding; therefore, the truncation strategy was maintained. A detailed breakdown of first-year events and the rationale for left truncation is provided in **Supplementary Material,** Section 3**.**

Nevertheless, several limitations warrant consideration. First, the retrospective design carries inherent risks of residual confounding, despite IPW adjustment. Second, the bovine cohort was relatively small (n = 37), limiting statistical power to detect modest inter-group differences. Moreover, it should be noted that nearly half of the bovine bioprostheses were Inspiris Resilia®, which have previously been associated with reduced durability. Third, MACE included composite events of moderate prosthetic deterioration, reintervention, or death; differences in indications for reintervention or threshold for intervention may vary between centers and over time. Finally, advances in surgical technique, postoperative care, and introduction of newer bioprosthetic designs over the 12-year accrual period may confound longitudinal comparisons.

The potential influence of antithrombotic/anticoagulation therapy on bioprosthetic valve durability has gained increasing attention, particularly in light of emerging data on subclinical leaflet thrombosis [7,28]. In our multicentre cohort, postoperative antithrombotic management was heterogeneous and evolved over the 12-year study period, reflecting the absence of specific guideline recommendations for bioprosthetic valves in the pulmonary position. Although aspirin was frequently prescribed for several months postoperatively, detailed longitudinal information was inconsistently available, limiting our ability to evaluate its potential protective effect. In addition, some patients received oral anticoagulation due to independent clinical indications—most commonly atrial fibrillation—which further contributed to variability in postoperative antithrombotic exposure. Future prospective studies with standardised antithrombotic protocols and systematic follow-up will be essential to clarify this aspect.

In light of our findings—demonstrating superior long-term durability of porcine bioprostheses once early postoperative events are excluded—and the broader body of literature, prosthesis selection for PVR should continue to be guided by individual patient anatomy, anticipated future transcatheter needs, and surgeon experience. Although both materials provide acceptable mid- and long-term performance, our truncated analysis suggests that porcine valves may offer a clinically meaningful advantage in the phase where structural valve degeneration becomes relevant. At the same time, newer-generation bovine pericardial valves incorporating anticalcification technologies, as well as evolving porcine tissue processing methods, warrant continued comparative evaluation. Prospective studies—ideally randomized—or large-scale registry linkages incorporating device-specific hemodynamic and infection data will be essential to refine prosthesis selection algorithms for the growing adult congenital heart disease population.

## 5 Conclusions

In this multicenter cohort with long-term follow-up, both bovine pericardial and porcine bioprosthetic valves demonstrated acceptable performance after surgical pulmonary valve replacement. However, when early postoperative events were excluded and baseline characteristics were balanced through inverse probability weighting, porcine bioprostheses showed a lower adjusted hazard of MACE during mid- and long-term follow-up. These findings suggest that material-related differences in durability may become more apparent beyond the first postoperative year. While prosthesis selection should continue to be guided by patient anatomy, anticipated future interventions, and surgical considerations, our results support the need for ongoing comparative evaluation of bioprosthetic materials and for prospective studies that further clarify their long-term performance in the adult congenital heart disease population.

## Declaration of generative AI and AI-assisted technologies in the manuscript preparation process

During the preparation of this work the author(s) used Microsoft Copilot in order to improve the clarity, fluency, and precision of the English language. After using this tool/service, the author(s) reviewed and edited the content as needed and take(s) full responsibility for the content of the published article.

## CRediT authorship contribution statement

**Eliú David Pérez Nogales:** Conceptualization, Data curation, Formal analysis, Investigation, Methodology, Project administration, Writing – original draft, Writing – review & editing. **Elisabet Viera Reyes:** Data curation, Methodology, Supervision, Writing – review & editing. **Stefano Urso:** Conceptualization, Investigation, Methodology, Project administration, Writing – review & editing. **María Luz Polo López:** Data curation, Writing – review & editing. **Blanca Torres Maestro:** Data curation, Investigation, Methodology, Writing – review & editing. **Jesús María González-Martín:** Formal analysis, Methodology. **Pablo Meras Colunga:** Conceptualization, Writing – review & editing. **Jose Ruíz Cantador:** Methodology, Supervision, Writing – review & editing. **Carlos Merino Argos:** Data curation, Investigation, Writing – review & editing.

## Declaration of competing interest

The authors declare that they have no known competing financial interests or personal relationships that could have appeared to influence the work reported in this paper.
