# Supplementary material for: Bovine pericardial versus porcine bioprosthetic valves in pulmonary position for adult congenital heart disease: A three-center, retrospective, propensity-weighted analysis of clinical outcomes
Source: Int J Cardiol Congenit Heart Dis. 2026 Aug 5;25:100700. doi: 10.1016/j.ijcchd.2026.100700 (PMC13524727; doi:10.1016/j.ijcchd.2026.100700)
Supplement: Supplementary file 1 [file mmc1.docx]

**Title: Bovine pericardial versus porcine bioprosthetic valves in pulmonary position for adult congenital heart disease: A three-center, retrospective, propensity-weighted analysis of clinical outcomes.**

**SUPPLEMENTARY MATERIAL**

**Supplementary material. Section 1. Survival analysis by prosthesis size. The critical role of the propensity score.**

Across the entire cohort, a substantial proportion of MACE events occurred in patients implanted with smaller prosthetic valves (<25 mm). This pattern is consistent with the well‑established observation that **smaller bioprostheses degenerate more rapidly**, largely due to higher leaflet stress, reduced effective orifice area, and accelerated structural valve deterioration (younger, smaller and stronger immune system).

**In the porcine group,** **5 of the 37 MACE events** occurred in patients implanted with valves below 25 mm (three 23‑mm valves and two 21‑mm valves). All five patients weighed under 30 kg, which is physiologically coherent: smaller body size necessitates smaller prostheses, and smaller prostheses are known to experience higher mechanical stress and earlier degeneration. **In the bovine group, 3 of the 7 MACE events occurred in valves below 25 mm** (two 23‑mm valves and one 21‑mm valve). Again, all except one case involved patients under 30 kg.

Kaplan–Meier analysis demonstrated that smaller prostheses (<25 mm) had significantly lower durability, with a highly significant difference: Overall cohort: p = 0.0059 and porcine valves: p = 0.0031 (clear separation of curves, better survival for ≥25 mm). Bovine valves: not statistically significant, likely due to limited sample size, but the trend was directionally consistent. This reinforces that prosthesis size is one of the strongest determinants of bioprosthetic durability, and smaller valves are consistently associated with earlier structural valve deterioration. **Supplementary material. Figure 1.**

Why this matters for the propensity score: Because prosthesis size is such a powerful predictor of durability, it is essential to adjust for it when comparing materials. This is precisely why the propensity score is crucial, it balances the distribution of valve sizes between groups, ensuring that any observed difference in durability is attributable to material, not to systematic differences in prosthesis size.

**Supplementary material. Figure 1.** Distribution of MACE events according to prosthesis size (<25 mm vs ≥25 mm) across the entire cohort (A) and stratified by bovine (B) and porcine (C) bioprostheses. Kaplan–Meier curves demonstrated significantly lower durability for smaller prostheses in the overall cohort (p = 0.0059) and in porcine valves (p = 0.0031), with a similar but non‑significant trend in bovine valves, likely reflecting limited sample size.

**Supplementary material. Section 2. Evaluating MACE Risk in Inspiris vs. Non‑Inspiris Bovine Valves.**

Regarding the proportion of MACE occurring in Inspiris bovine valves, we examined this carefully given the growing concern in the literature about early failure of Inspiris prostheses in the pulmonary position. In our cohort of 37 bovine valves, 17 were Inspiris (45.9%). Among the MACE events, 3 of the 17 Inspiris valves (17.65%) experienced MACE, compared with 4 events among the 20 non‑Inspiris valves (20%). Therefore, in our dataset, the difference in event rates between Inspiris and non‑Inspiris bovine prostheses was not as marked as previously reported, and did not suggest a disproportionate risk associated with the Inspiris model. This contrasts with published concerns regarding the durability of Inspiris valves in the pulmonary position, where early structural valve deterioration has been described more prominently. **It is possible that this discrepancy is related to the relatively small sample size of Inspiris valves in our cohort, and that a larger number of cases might reproduce the same conclusions reported in the literature. Furthermore, although we initially considered excluding Inspiris valves from the analysis due to these concerns, the absence of a higher MACE rate compared with the remaining bovine prostheses led us to retain them in the study to avoid introducing selection bias.**

**Supplementary material. Section 3. First-year truncated patients and rationale for the one-year left truncation.**

The decision to apply left truncation at one year was made a priori, before reviewing the causes of events occurring during this period, with the explicit aim of isolating prosthesis-related durability from early postoperative complications unrelated to the valve itself. This methodological approach is consistent with the VARC-3 consensus framework, which distinguishes between periprocedural mortality (≤30 days or index hospitalization), early mortality (>30 days to 1 year), and late mortality (>1 year), recognizing that events within the first year predominantly reflect procedural safety and patient-related factors rather than bioprosthetic valve performance [1]. The VARC-3 document further recommends that the assessment of bioprosthetic valve durability — including structural valve deterioration and bioprosthetic valve failure — should focus on the period beyond the early postoperative phase, as early events are more likely attributable to surgical technique, perioperative complications, or pre-existing comorbidities than to intrinsic prosthesis degeneration.

Importantly, the one-year truncation point was selected before examining the individual causes of events during this period, specifically to avoid introducing selection bias by choosing a truncation point that would favor one valve type over another. The causes of the 8 MACE events occurring within the first year were reviewed only after the primary analysis was completed and are reported here for transparency.

Of the 8 patients who experienced MACE during the first year:

- **In-hospital deaths (n = 4):**
  1. Two gastrointestinal hemorrhage
  2. Severe hepatic insufficiency secondary to pre-existing liver cirrhosis, decompensated after surgery
  3. Postoperative respiratory distress with refractory ventilatory failure
- **Post-discharge deaths (n = 2):**
  5. Lung cancer (previously known at the time of valve implantation)
  6. Cholangiocarcnoma
- **Prosthesis-related event (n = 2):**
  8. Prosthetic valve failure.

As shown, 6 of the 8 first-year events were clearly unrelated to prosthesis performance, being attributable to perioperative surgical complications (respiratory distress, gastrointestinal hemorrhage), pre-existing comorbidities (liver cirrhosis, known lung cancer) or newly diagnosed malignancy. Only 2 event represented true prosthetic valve failure. This distribution strongly supports the rationale for left truncation: including these events in the primary analysis would have contaminated the assessment of prosthesis-related durability with outcomes driven by surgical risk and patient comorbidities rather than by the intrinsic performance of the bioprosthesis.

All 8 MACE events occurring within the first year were observed in the porcine prosthesis group. This finding warrants careful interpretation. First, the porcine group comprised 84% of the total cohort (n = 194 vs. n = 37 for bovine pericardial), meaning that low-incidence events — such as perioperative multiorgan failure, gastrointestinal hemorrhage, or concurrent malignancy — are statistically more likely to be captured in the larger group simply by virtue of sample size. In a cohort with such marked asymmetry, the absence of rare events in the smaller group does not imply a protective effect but rather reflects insufficient statistical power to detect them. Second, surgeon familiarity for some porcine prostheses may have introduced a channeling bias whereby more complex patients — those with more advanced congenital heart disease, prior surgical interventions, or significant comorbidities conferring higher perioperative risk — were preferentially assigned porcine valves. This selection pattern, driven by greater surgical experience with a specific prosthesis type in technically demanding cases, would concentrate perioperative complications in the porcine group irrespective of valve performance.

for greater transparency, we also performed survival analyses using the full follow‑up period and applying inverse probability weighting after propensity score adjustment. Although this analysis did not reach statistical significance (p = 0.119), the Kaplan–Meier curves show a clear and consistent trend toward better long‑term performance of porcine prostheses, with visible separation of the curves over time. **Supplementary material. Figure 2.** This complementary analysis reinforces the main findings and provides an unbiased depiction of the cohort’s behavior beyond the first‑year truncation.

**Supplementary material. Figure 2.** Kaplan–Meier curves for freedom from MACE using full follow‑up and inverse probability weighting after propensity score adjustment. Although the comparison does not reach statistical significance (p = 0.119), the curves show a consistent trend toward improved long‑term performance of porcine bioprostheses, with progressive separation over time.

**Supplementary Material, Section 4. Propensity Score.**

**Supplementary material. Figures 3 and 4:** Propensity scores for treatment groups (1). Standardized mean differences before and after IPW-adjustments (2). iEAO: indexed to body surface effective orifice area. BMI: Body mass index. ToF: Tetralofy of Fallot.

**Supplementary references:**

[1]	VARC-3 WRITING COMMITTEE:, Généreux P, Piazza N, Alu MC, Nazif T, Hahn RT, et al. Valve Academic Research Consortium 3: Updated Endpoint Definitions for Aortic Valve Clinical Research. JACC 2021;77:2717–46. https://doi.org/10.1016/j.jacc.2021.02.038.
